# *Roseicella aerolata* GB24^T^ from bioaerosol attenuates *Streptococcus pneumoniae*-introduced inflammation through regulation of gut microbiota and acetic acid

**DOI:** 10.3389/fmicb.2023.1225548

**Published:** 2023-07-20

**Authors:** Tian Qin, Ting Yu, Yuqi Liu, Jiguo Wu, Yunxia Jiang, Guoxia Zhang

**Affiliations:** ^1^NMPA Key Laboratory for Safety Evaluation of Cosmetics, Guangdong Provincial Key Laboratory of Tropical Disease Research, Department of Environmental Health, School of Public Health, Southern Medical University, Guangzhou, China; ^2^Guangdong-Hong Kong-Macao Joint Laboratory for Contaminants Exposure and Health, Guangzhou, China

**Keywords:** *Streptococcus pneumoniae* infection, *Roseicella aerolata* GB24^T^, gut–lung axis, short-chain fatty acids, acetic acid

## Abstract

*Streptococcus pneumoniae* (*Spn*) is the most common respiratory pathogen causing community-acquired pneumonia. Probiotics represent a new intervention target for *Spn* infection. Hence, the discovery and development of new potential probiotic strains are urgently needed. This study was designed to investigate the beneficial effect and mechanism of a new bacterium named *Roseicella aerolata* GB24^T^ that antagonizes *Spn* at cellular and animal levels. The results revealed that GB24^T^ strain inhibited the growth of *Spn* on sheep blood agar plates, forming inhibition circles with a diameter of 20 mm. In cultured bronchial epithelium transformed with Ad 12-SV40 2B (BEAS-2B) cells, *Spn* infection induced an elevation in the expression levels of interleukin-1β, interleukin-6, and tumor necrosis factor-α to 4.289 ± 0.709, 5.587 ± 2.670, and 5.212 ± 0.772 folds compared to healthy controls, respectively. Moreover, pre-infection with GB24^T^ for 1.5 h almost eliminated the cellular inflammation caused by *Spn* infection. Additionally, male Sprague–Dawley rats infected with *Spn* were randomly allocated into two groups: GB24^T^ pre-infection and *Spn* infection groups, with healthy rats as control. GB24^T^ significantly alleviated inflammatory lung injury caused by *Spn* infection, which was associated with obvious changes in the abundance of gut microbiota and a trend toward enhanced secretion of short-chain fatty acids, especially acetic acid. Acetic acid was validated to be effective in alleviating inflammation due to *Spn* infection in cellular assays. Together, these findings highlight that GB24^T^ strain is an important protective feature in the respiratory tract.

## 1. Introduction

The incidence and hospitalization for community-acquired pneumonia (CAP) are high in China, the United States, and Europe (Torres et al., 2013; Jain et al., 2015; Sun et al., 2020). *Streptococcus pneumoniae* (*Spn*) is the most predominant reason for bacterial CAP in all ages (Mandell et al., 2007). Pneumococcal conjugate vaccines (PCV) are effective against invasive pneumococcal disease (IPD) caused by selected drug-resistant serotypes (Fitzgerald and Waterer, 2019). For example, 7-valent (PCV7) and 13-valent (PCV13) pneumococcal conjugate vaccination resulted in a 91% reduction in the incidence of IPD in the United States in 2019 compared with 1997 (Wasserman et al., 2021). Nevertheless, the validity of conventional vaccines continues to deteriorate due to the phenomenon of serotype substitution, i.e., the increase in invasive diseases caused by non-vaccine strains (Briles et al., 2019). At present, antibiotics are still the primary treatment for pneumonia caused by *Spn* infection (Olson and Davis, 2020). There is a remarkably high incidence of multi-drug resistance (MDR, 50.8%) and resistance against macrolides antibiotics (MA, 51.6–66.8%) of *Spn* in Asia (Kim et al., 2020). Conversely, the development of a new antibiotic would take a remarkably long time with high financial costs (Fu et al., 2023). Thus, there is an urgent need to develop alternative approaches for preventing or treating *Spn* infections.

Beneficial strains have been proven as an effective measure of antagonism against the pathogen. A probiotic supplement can be gradually applied as a new intervention method to prevent and treat respiratory infectious diseases such as *Spn* infection. For example, nasal inhalation of live *Bifidobacterium*, *Lactobacillus*, *Enterococcus*, and *Bacillus* (CBLEB) can effectively protect immunocompromised rat models of *Spn* infection (Lv et al., 2022). *L. casei* CRL431 and *L. rhamnosus* CRL1505 can modulate a range of inflammatory responses and lung damage caused by *Spn* infection (Barbieri et al., 2017; Zelaya et al., 2020). Undoubtedly, microbial therapy is expected to become a reliable means to prevent and treat *Spn* infection. *Roseicella* is a member of the family *Acetobacteraceae*, and no *Roseicella* strain has been found to be pathogenic. *Rosecella aerolata* GB24^T^ is a novel strain isolated from bioaerosol, with no virulence factor in its whole genome. As a consequence, such microorganisms are an excellent protection option as a highly effective antagonistic strain against *S. pneumoniae*.

The increasing sophistication of high-throughput sequencing technologies has helped us gain a more convenient and comprehensive understanding of the gut microbial community. Being the largest microbiota-colonizing environment in the human body, the gut is colonized with about 10^14^ bacteria (Hillman et al., 2017; Chen et al., 2021). Moreover, various studies have indicated that there is interference between gut microbiota and the lung, called the gut–lung axis (Tan et al., 2020), meaning that the changes in gut microbiota composition are sensitive to the health status of the lung. Therefore, the gut microbiota offers us a novel perspective to prevent respiratory diseases.

As important fermentation products of intestinal bacteria, short-chain fatty acids (SCFAs) are closely related to the inflammatory response in the body. SCFAs play an important role in the anti-inflammatory and immune regulation of the gut–lung axis (Rastogi et al., 2022), such as promoting the differentiation of helper T cells, stimulating the expression of cytokines, and inhibiting the occurrence of allergic airway diseases (Arpaia et al., 2013; Park et al., 2015). Hence, SCFAs have application potential in preventing and treating respiratory diseases.

We hypothesized that the new GB24^T^ strain isolated from bioaerosols can reduce inflammation induced by *S. pneumoniae* infection, and the gut microbiota and SCFAs might be important participants in this process. Therefore, we systematically evaluated the role of GB24^T^ strain in the inflammatory response induced by *Spn* infection at both cellular and animal levels to test the hypothesis and understand the action mechanism of GB24^T^ strain. In parallel, we attempted to clarify the influence of gut microbiota and identify key bacteria metabolites. We aimed to better our exploring of the effect of the GB24^T^ strain on attenuating *Spn* infection by regulating the gut microbiota structure and metabolic functions.

## 2. Materials and methods

### 2.1. Bacterial strains, cells, and rats

The GB24^T^ strain was isolated from bioaerosols of a typical e-waste dismantling site in South China (23.32° N, 116.36°1 E) in November 2019 (Pan et al., 2021). Based on the results of biochemical characterization, 16S rRNA, and genomic comparison, GB24^T^ was classified as a new species of *Roseicella* (Qin et al., 2022). GB24^T^ was cultured aerobically at 37°C in a nutrient broth medium (Beijing Solarbio Science & Technology Co., Ltd.). *Spn* ATCC 49619 (serotype 19F) and BEAS-2B cells ATCC CRL-9609 were purchased from the American-type culture collection (ATCC). *Spn* was aerobically cultured in a 5% sheep blood agar plate (Guangdong Huan Kai Microbial Technology Co., Ltd.) at 37°C, while BEAS-2B cells were cultured in Dulbecco’s modified eagle medium [DMEM, high glucose, ThermoFisher Biochemical products (Beijing) Co., Ltd.] with 10% fetal bovine serum [ThermoFisher Biochemical products (Beijing) Co., Ltd.] and 1% antibiotics (100 units × mL^−1^ penicillin and 100 mg × mL^−1^ streptomycin, Beijing Solarbio Science & Technology Co., Ltd.) at 37°C and 5% CO_2_. This randomized trial used 36 male Sprague–Dawley rats (8 weeks, 180–220 g, Specific pathogen free) purchased from the Southern Medical University Experimental Animal Center. The experimental animal ethics committee of Southern Medical University approved this study (SMUL2021099). All rats were housed in relatively constant temperature chambers with 12-h circulating light, adequate drinking water (tap water), and standard chow (Beijing Keao Xieli Feed Co., Ltd.). The experimental procedures complied with the relevant national requirements and regulations regarding the ethics of animal experiments.

### 2.2. Experimental treatments

#### 2.2.1. Treatments of strains and cells

Sheep blood plates were utilized to resuscitate *Spn*, followed by the culture at 37°C. The plates were rinsed daily with sterile phosphate-buffered saline (PBS, Beijing Solarbio Science & Technology Co., Ltd.) and then coated in appropriate amounts on a new blood plate for passaging. At least two passages were made for subsequent experiments. Then, 200 μL overnight cultured *Spn* (OD_600_ = 0.5) solution was taken and spread uniformly on the blood plate. GB24^T^ was immediately coated using an inoculating loop to an approximately 5-mm diameter cultivation circle on the same blood plate. The diameters of *Spn* inhibition circles were recorded.

BEAS-2B cells were seeded at a density of 5 × 10^3^ cells per well into 96-well plates and cultured overnight at 37°C and 5% CO_2_. After the cells fully adhered to the wall, the cells were infected with various doses of *Spn* (MOI = 1, 20, 50, and 100) for 1.0, 1.5, 2.0, 3.0, 6.0, and 9.0 h or GB24^T^ (MOI = 1, 10, 20, 30, 40, and 50) for 1.5 h. We then added 110 μL of CCK-8 working solution CCK-8 primitive liquid (Shanghai Beyotime Biotechnology Co., Ltd.) to the DMEM medium (with 10% FBS) = 10: 100 per well after washing the cells three times with sterile 4°C pre-cooled PBS and cultured them for 2.0 h at 37°C and 5% CO_2_. The absorbance was measured at 450 nm with a microplate reader [ThermoFisher Biochemical Products (Beijing) Co., Ltd.]. Moreover, BEAS-2B cells were seeded at a density of 1 × 10^6^ cells per well into six-well plates and cultured overnight at 37°C and 5% CO_2_. The cells were divided into four groups: control (Con), *Spn*, GB24^T^: *Spn* = 1:1 (1:1), and GB24^T^: *Spn* = 2:1 (2:1). As shown in Supplementary Table S1, the cells were infected with different doses of GB24^T^ or *Spn*. The vehicle for GB24^T^ and *Spn* was the DMEM medium (with 10% FBS).

#### 2.2.2. Treatments of animals

**Figure 1:**
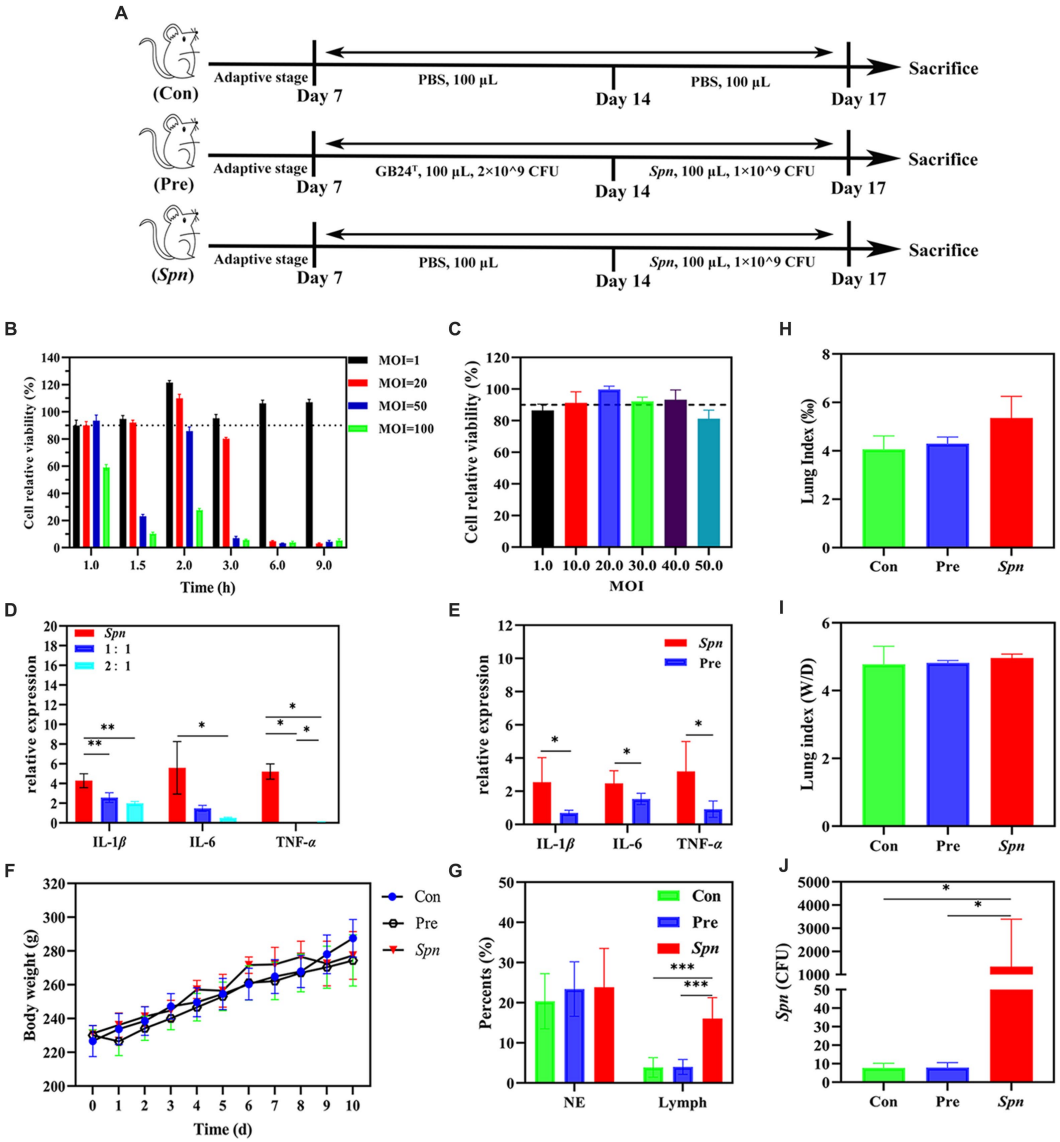
Protective effect of GB24^T^ against *Spn* infection. **(A)** Details of animal experiments: No treatment was done for the first 7 days. Rats were dissected on 8–14 days with daily nasal drops of GB24^T^ or sterile PBS and on days 15–17 with daily drops of *Spn* or sterile PBS and dissected on day 18. Cellular activity of BEAS-2B cells infected with different doses of *Spn* **(B)** or GB24^T^ **(C)** at various durations. Relative expression levels of inflammatory cytokines in BEAS-2B cells **(D)** and rat lungs **(E)**. Body weight changes **(F)**, Leukocyte ratio in BALF **(G)**, Lung index (lung weight/body weight × 1,000%) **(H)**, and lung wet-dry weight ratio (Wet weight /dry weight) **(I)**. The settlement quantity of *Spn* in the lungs was examined using qPCR **(J)**. *p*-values: * < 0.05, ** < 0.01, and *** < 0.001 indicate statistically significant differences between groups.

Rats were randomly divided into three groups: healthy controls (Con), GB24^T^ pre-infected rats (Pre), and *Spn*-infected rats (*Spn*), and each group consisted of 12 rats. As shown in Figure 1A, all rats were experimentally treated after 7 days of acclimatization feeding. Briefly, the rats were anesthetized by the nasal inhalation of ether in a closed environment with air isolation, and then *Spn*, GB24^T^, or PBS was administered *via* nasal drops. All rats were sacrificed after intraperitoneal anesthesia with chloral hydrate (13%, m/v, 0.35 mL × 100 g^−1^), followed by blood collection from the abdominal aorta on day 18. Feces were collected for the last 3 days and immediately stored at −80°C. Blood was centrifuged at 3500 rpm at 4°C to extract serum (1 mL) and stored at −80°C along with the collected lung tissue, small intestine, liver, cecum, and its contents.

### 2.3. Lung indicators

#### 2.3.1. Pulmonary phenotype, lung index, and the wet-dry weight ratio of lung

After blood collection from the abdominal aorta, the chest cavity was opened immediately to expose the intact lung, and the front and back of the lung were photographed. The lungs were weighed as soon as possible after absorbing the water on the surface with gauze and then dried in an oven at 60°C for 5 days before being weighed again. Calculation of pulmonary organ coefficient (Lung weight/Bodyweight) and wet-dry weight ratio for the evaluation of edema using Microsoft Office Excel (2016).

#### 2.3.2. Bronchoalveolar lavage fluid

BALF of rats was collected following the method of Fu et al. (2019). After exposing the rat’s trachea, 9 mL of sterile pre-cooled PBS was injected into the trachea in three separate injections using a 5-mL disposable syringe. The syringe was pushed and aspirated back and forth three times and then recovered into a sterile centrifuge tube. The BALF was allowed to stand for 1 h and then observed visually for the cell precipitation and its color. BALF was centrifuged at 2000 rpm and 4°C for 10 min and mixed in 1 mL of pre-cooled sterile PBS. The cell suspension (20 μL) was taken to a hemocytometer plate to count white and red blood cells separately and calculate their ratio. One drop of cell suspension was taken for smearing, fixed in methanol for 20 min, and then stained according to the instructions of the Diff-Quick stain kit (Beijing Solarbio Science & Technology Co., Ltd.).

#### 2.3.3. Hematoxylin–eosin staining and scoring

The right middle lobe of the lung of three rats in each group was randomly selected, fixed in 4% paraformaldehyde for 48 h, and then cut into lung tissue sections (4-μm thick). HE staining of the sections was conducted following the instructions of the HE staining kit (Biosci Biotechnology Co.; Ltd.) to visualize the microscopic condition of the lung. Next, we used an orthomosaic (Olympus China Ltd.) microscope and its imaging system (Hamamatsu, Japan) to photograph the surface of the sections at different locations and analyzed them using NDP.view2. Finally, histopathology scores of the lung were calculated under double-blind conditions. The specific scoring criteria are shown in Supplementary Table S2.

#### 2.3.4. Quantitative detection of *Spn* invasion

DNA was extracted from *Spn* by the phenol-chloroform method. An ultra-micro spectrophotometer K5500 (Beijing KRIRO Science and Technology Development Co., Ltd.) was used to monitor the concentration and purity of the DNA. Expression of lytA in the lung of rats was checked using real-time quantitative PCR (qPCR) on the concentration of DNA at 1, 10^−1^, 10^−2^, 10^−3^, 10^−4^, and 10^−5^ μg × μL^−1^. A scatter plot showing the concentration of DNA (C_DNA_) and qPCR cycle times (Ct) was acquired (Supplementary Figure S1). The curve equation was Y = -3.2276x + 10.5500, *R^2^* = 0.9966. log_10_ C_DNA_ and Ct values were represented by x and y, respectively. Based on the data from Dunne (Dunne et al., 2014), we summarized the quantitative relationship between the quantity of *Spn* and Ct values as Y = −3.2276x + 38.4710, *R^2^* = 0.9966. log_10_ CFU and Ct values were represented by x and Y, respectively.

### 2.4. RNA isolation and qPCR

Tissues of the lung (0.05 g) were homogenized using an organizational homogenizer (Shanghai Fluke Technology Development Co., Ltd.). BEAS-2B cells were fully raged in RNAex Pro Reagent (Hunan Aikerui Biological Engineering Co., Ltd.). The concentration and purity of the RNA were measured using an ultra-micro spectrophotometer K5500. RNA (500 ng) was reverse transcribed into complementary DNA (cDNA) using *Evo M-MLV* reverse transcription pre-mix (Guangzhou Ruizhen Biology Technology Co., Ltd.) and PCR instrument (Bio-Rad). qPCR was performed using the SYBR green PCR kit (Guangzhou Ruizhen Biology Technology Co., Ltd.) and the QuantStudioTM 6 flex qPCR system (Thermo Fisher Scientific). The result was visualized using QuantStudio™ qPCR software. The expressions of mRNA were calculated using the comparative cycle threshold (Ct) method and normalized to glyceraldehyde-3-phosphate dehydrogenase (GAPDH) in the same cDNA samples. Sequences of primers are listed in Supplementary Table S3.

### 2.5. Gut microbiota profiling

Six fecal samples (0.05 g) were randomly selected from each group for high-throughput sequencing of 16S rRNA gene. Sequencing service was provided by Wekemo Tech Group Co., Ltd. Shenzhen China. The raw sequence data were uploaded to the National Center for Biotechnology Information Sequence Read Archive SRA database (PRJNA908648) and analyzed on the free online platform of Wekemo Bioincloud.

### 2.6. Short-chain fatty acids extraction and measurement

Fecal samples from six rats in each group were randomly selected for the determination of SCFAs. Two sets of feces (0.5 g each) were weighed and placed in two sterile 2-mL centrifuge tubes in advance. We added 0.5 mL of pre-chilled PBS to each tube, vortexed and shook them for 10 s, and then sonicated them in an ultrasonic cleaner for 10 min. The supernatant was then collected by centrifugation at 13,000 rpm and 4°C for 10 min into 2-mL centrifuge tubes. H_2_SO_4_ (10 μL; 50%) solution was added to the tubes that were dried with 0.5 g calcium chloride to absorb water in advance. Subsequently, 1 mL of ether was added to the centrifuge tube, vortexed, and centrifuged at 6000 rpm and 4°C for 10 min. Finally, 10 μL of 2-ethyl butyric acid ethyl ether solution (250 μg × mL^−1^) was added to the supernatant as an internal standard and analyzed by gas chromatography–mass spectrometry (GC–MS) as soon as possible. Additionally, GB24^T^ in a logarithmic growth phase was assessed, and the composition of SCFAs was detected after rejecting the bacteria and adding the internal standard.

### 2.7. Anti-inflammatory capacity of acetic acid

BEAS-2B cells were seeded at a density of 1 × 10^6^ cells per well into six-well plates and cultured overnight at 37°C and 5% CO_2_. The cells were stimulated with or without sodium acetate (0.1, 0.5, 1.0, 1.5, and 2.0 mM) for 1.5 h, followed by co-culture with *Spn* (1 × 10^7^ CFU × mL^−1^) for 2 h. CCK-8 and qPCR were used to evaluate the vitality of cells and detect the relative expression of interleukin-1β (IL-1β), IL-6, and tumor necrosis factor (TNF-α).

### 2.8. Statistical analysis

All data were presented as mean ± standard deviation of the mean (SEM) and analyzed using IBM SPSS Statistics 20.0. The Kolmogorov–Smirnov and Levene tests were used to detect whether the data obeyed a normal distribution and compare the difference in variance between the groups, respectively. One-way ANOVA and Kruskal–Wallis tests were performed for the analysis of data from different groups. LSD or SNK was used to make two-by-two comparisons between the groups. Dunnett’s T3 was used for two-way comparisons between the groups when the variance was uneven. GraphPad Prism v. 8.4.3, TBtools v. 1.089, and Wekemo Bioincloud^1^ were used to draw related graphics. *p*-values * < 0.05, ** < 0.01, and *** < 0.001 were considered significantly different.

## 3. Results

### 3.1. GB24^T^ relieves inflammation caused by *Spn* infection

At the end of the co-culture with *Spn*, the formation of an inhibitory ring with a 20-mm diameter was observed around GB24^T^, indicating that GB24^T^ was able to inhibit the growth of *Spn*. As shown in Figures 1B,C, the viability of BEAS-2B cells was at a high level (> 90%) after 2.0 h (110.0% ± 2.9%) of *Spn* infection (MOI = 20) or 1.5 h (99.7% ± 2.2 and 93.2% ± 6.3%) of GB24^T^ (MOI = 20 and 40) infection. Therefore, the above-mentioned concentrations and staining times were used for subsequent experiments. The results of qPCR (Figure 1D) revealed that *Spn* infection increased the relative expression of IL-1β, IL-6, and TNF-α in BEAS-2B cells compared to healthy control by 4.289 ± 0.709, 5.587 ± 2.670, and 5.211 ± 0.772 folds, respectively. In contrast, GB24^T^ intervention (GB24^T^: *Spn* = 2:1) significantly reversed the elevated expression levels of the cellular inflammatory factors (*p* < 0.05). Moreover, consistent with the results of cellular experiments, *Spn* infection raised the gene expression levels of IL-1β, IL-6, and TNF-α in the rat’s lungs to 2.541 ± 1.486, 2.475 ± 0.756, and 3.197 ± 1.795 folds compared to the Con group, respectively, and GB24^T^ intervention caused a reversal of the elevated expression levels of cellular inflammatory factors (*p* < 0.05; Figure 1E).

A decreasing trend of body weight was observed after *Spn* infection, and the trend was relieved after the intake of GB24^T^ (Figure 1F). Diff staining revealed no significant difference in the composition of neutrophils and lymphocytes in the BALF of rats in each group (Figure 1G). Moreover, the lung index (5.355 ± 0.892) and the wet/dry weight ratio (4.965 ± 0.113) of the rats in the *Spn* group showed a tendency to increase compared with those of the Con group (4.060 ± 0.559 and 4.780 ± 0.527; Figures 1H,I), but the trend was not statistically significant. The median *Spn* colonization density in the lungs of the *Spn* group was as high as 358.125 CFU/500 ng DNA, which was significantly higher than that in the other two groups (*p* < 0.01; Figure 1J).

### 3.2. Microstructural changes in rat lungs

**Figure 2:**
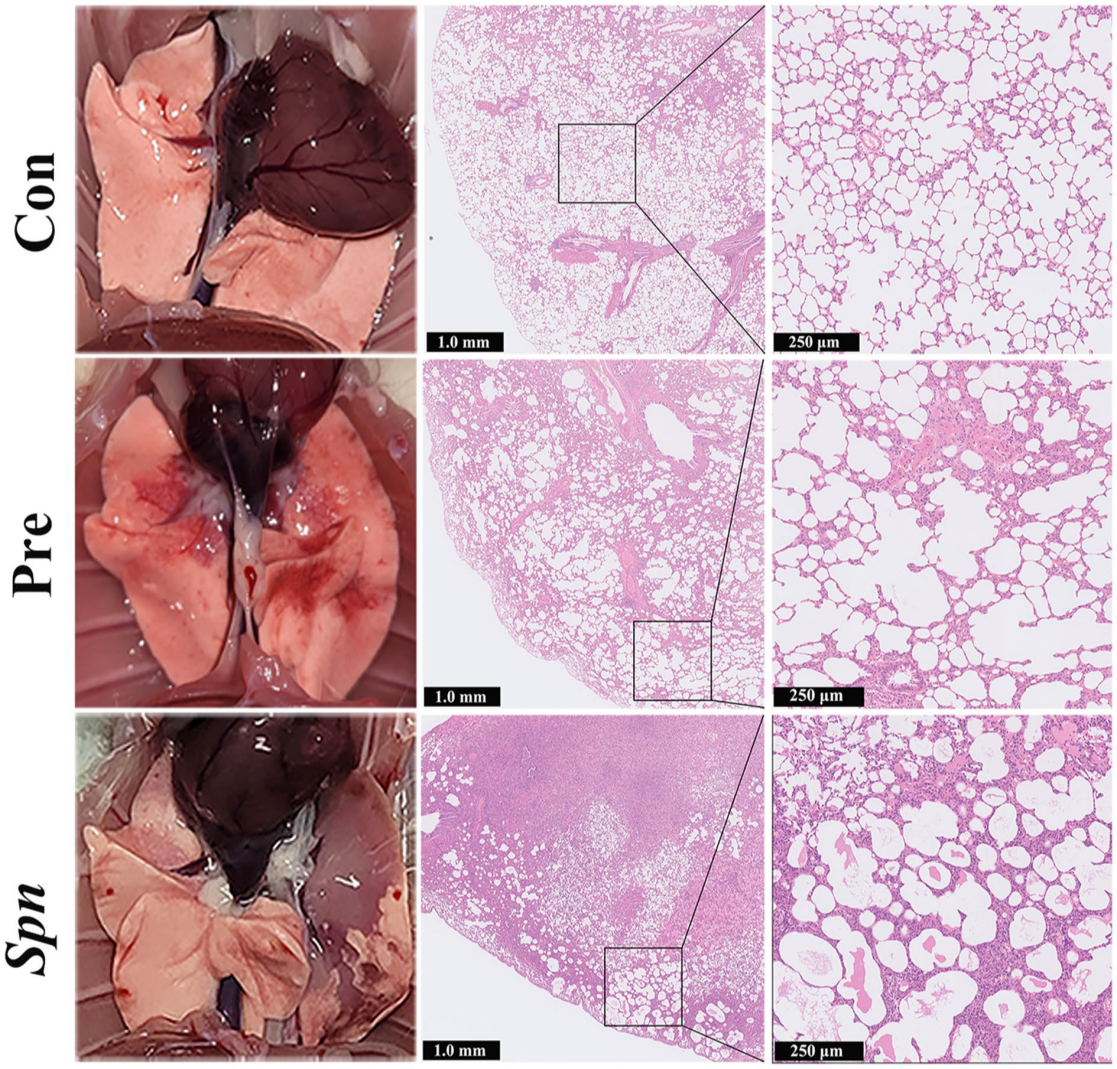
Apparent changes in lung tissue and HE-stained sections.

*Spn* infection led to visible congestion, petechiae, and tissue erosion in the lung tissue of rats, and the above-mentioned lesions were significantly alleviated in the Pre group. Furthermore, HE staining of histopathological sections from rats in the *Spn* group showed significant congestion, hemorrhage, inflammatory cell infiltration, and structural collapse in the alveolar cavity. Although alveolar wall thickening and inflammatory cells infiltration were also observed in the Pre group, the severity and area were significantly improved compared with the *Spn* group (Figure 2). Histopathological scoring results (Supplementary Figure S2) demonstrated that the total scores obtained in both *Spn* (11.00 ± 1.00, *p* < 0.001) and Pre (4.33 ± 0.58, *p* < 0.01) groups were significantly higher than those in the Con group (1.33 ± 0.58), while the Pre group obtained lower scores compared to the *Spn* group (*p* < 0.001).

### 3.3. Alterations in gut microbiota

#### 3.3.1. Changes in diversity and composition of gut microbiota

**Figure 3:**
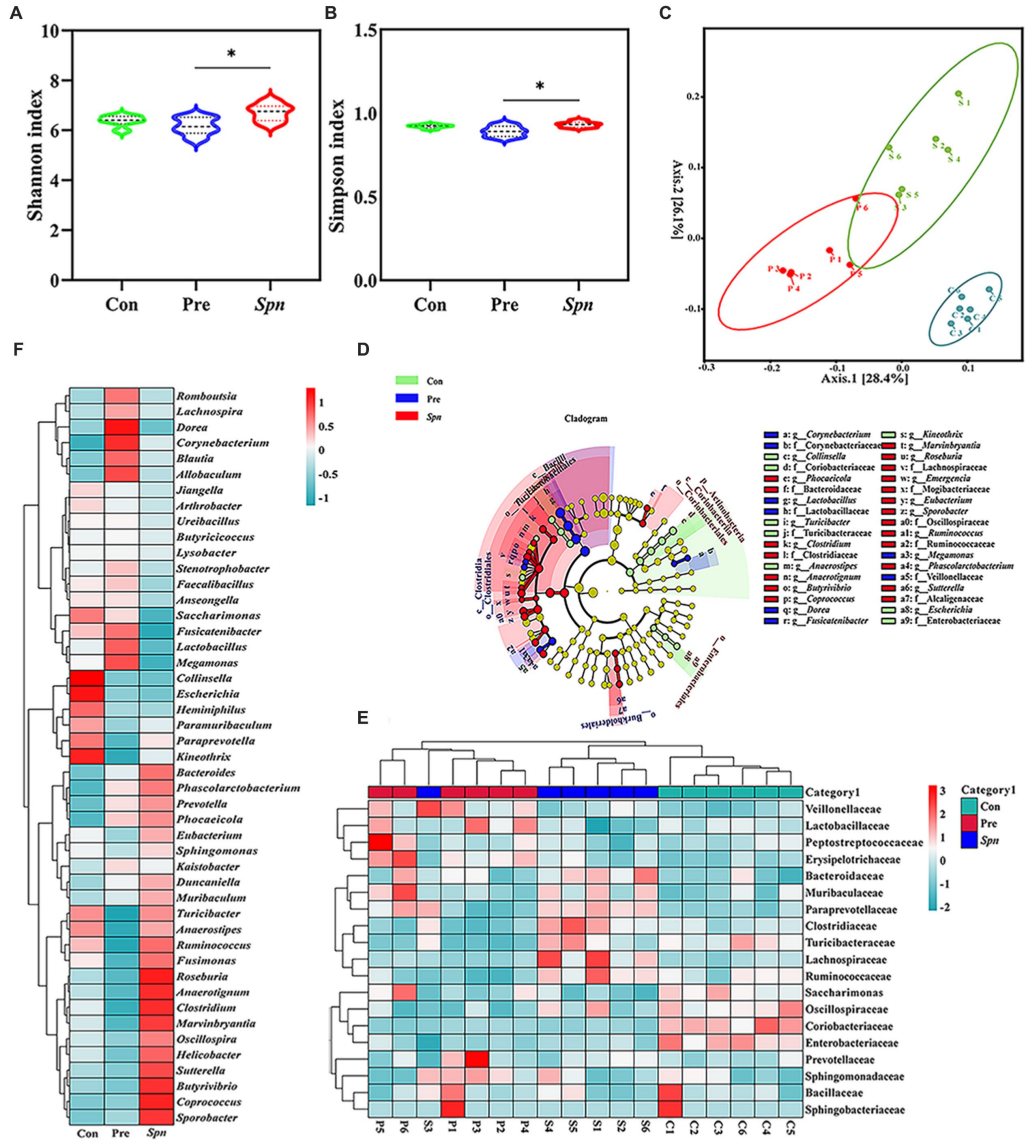
*α*-diversity index: Shannon **(A)** and Simpson index **(B)**. PCoA analysis based on Bray Curtis distance matrix **(C)**. Cladogram **(D)** for the species differing among groups, with an LDA Score of >3.0. The inside-out circle subscale represents the taxonomic level of species from kingdom to species, and the size of the circle diameter represents the relative abundance. The coloring principle: yellow represents no significant difference, all colors other than yellow represents species statistically different between groups, and species with the same color correspond to each other within the group. The normal standardized clustering heat map was constructed based on the top 19 families **(E)** and 47 genera **(F)** in terms of abundance. Red and blue represent higher and lower abundance, respectively. *p*-values: * < 0.05 indicate statistically significant differences between groups, *N* = 6.

A total of 8,717 bacterial operational taxonomic units (OTUs) (Supplementary Figure S3) were identified in high-throughput sequencing, of which 9.26% (806 OTUs) were shared among the three groups. As illustrated in Figures 3A,B, GB24^T^ nasal ingestion reduced the intestinal microbiota diversity in *Spn*-infected rats, with Shannon and Simpson indices decreasing from 6.71 ± 0.35 and 0.93 ± 0.02 in the *Spn* group to 6.16 ± 0.35 and 0.89 ± 0.03 (*p* < 0.05) in the Pre group. PCoA analysis based on the Bray–Curtis distance matrix indicated that the intestinal microbial composition of the Pre group was significantly dissimilar to that of Con and *Spn* groups (*p* < 0.05; Figure 3C), and LefSe analysis (LDA Score > 3.0) showed enrichment of 13 families and 23 genera among the groups, with *Lactobacillaceae* and *Lactobacillus* being significantly higher in the Pre group compared to the other two groups (*p* < 0.01; Figure 3D). At the level of phylum, Bacillota and Bacteroidota were the dominant phyla in the gut of rats in the three groups. Compared with the Con (71.1% ± 2.0, 25.2% ± 2.0%) and Pre (69.5% ± 4.3, 27.3% ± 4.5%) groups, the *Spn* group showed a decreasing and increasing trend in the relative abundance of Bacillota (67.0% ± 2.4%) and Bacteroidota (29.7% ± 2.7%), respectively, and the value of Bacillota / Bacteroidota also decreased (Supplementary Figure S4A).

At the family level, *Lactobacillaceae*, *Prevotellaceae*, *Muribaculaceae*, *Oscillospiraceae*, and *Lachnospiraceae* dominated the microbial profile in feces for all three groups (Supplementary Figure S4B). *Lactobacillaceae* (43.0% ± 5.3%) was significantly decreased in the *Spn* group compared to the Con (49.5% ± 2.2%; *p* < 0.05) and Pre (51.8% ± 4.5%) groups (*p* < 0.01). In contrast, *Lachnospiraceae* (9.5% ± 3.2%) was significantly increased compared to the Con (5.5% ± 0.8%) and Pre (5.0% ± 0.6%) groups (*p* < 0.01). The clustering heat map (Figure 3E) constructed with the top 19 families in terms of abundance also confirmed the above-discussed trend. Moreover, the clustering heat map (Figure 3F) and statistical analysis of the top 47 genera in terms of abundance indicated that *Spn* infection led to a significant addition of *Coprococcus*, *Sporobacter*, and *Phascolarctobacterium*, whereas the abundance of *Collinsella*, *Escherichia*, and *Butyrivibrio* declined at the genus level (*p* < 0.05). Notably, GB24^T^ nasal inhalation caused a significant increase (*p* < 0.05) in *Lactobacillus*, *Megamonas*, *Fusicatenibacter*, *Anaerotignum*, *Dorea*, *Butyrivibrio* and a decrease in *Coprococcus*, *Roseburia*, *Turicibacter*, *Clostridium*, *Sutterella*, *Ruminococcus*, *Anaerostipes*, *Marvinbryantia*, and *Helicobacter* compared to the *Spn* group (*p* < 0.05).

#### 3.3.2. Correlation of lung inflammation levels, SCFAs, and gut microbiota

**Figure 4:**
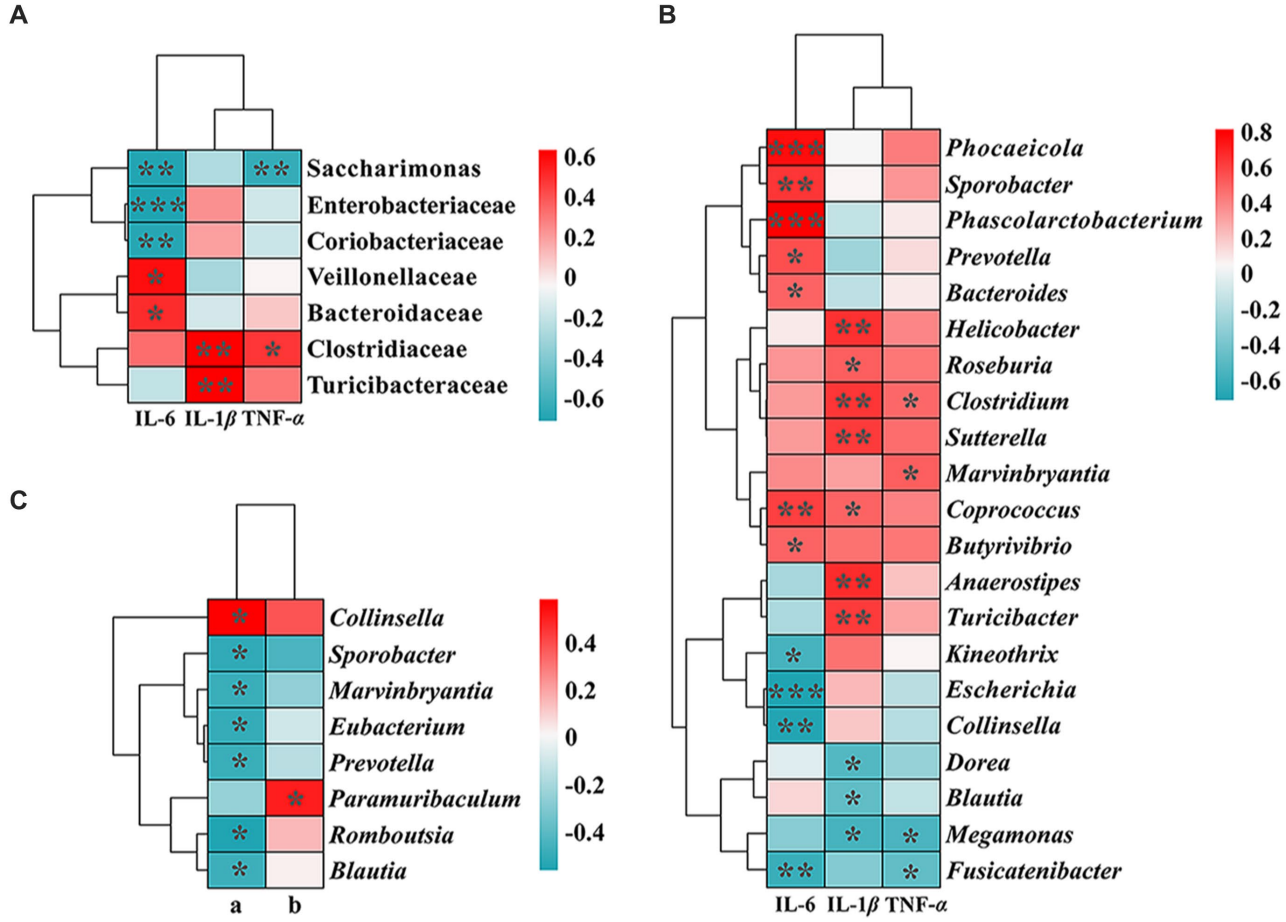
Correlation heat map between inflammatory cytokines **(A)**, SCFAs: a, acetic acid; b, butyric acid. **(B,C)**, and the abundance of gut microbiota. The correlation coefficient threshold was set to 0.4. The *p*-values * < 0.05, ** < 0.01, and *** < 0.001 indicate significant differences between groups.

A Spearman correlation analysis of inflammatory cytokine expression levels and two differential SCFAs with the top 19 bacterial families and 47 genera in number were performed, followed by constructing correlation heat maps (Figure 4), to further explore the relationship between intestinal microbiota, organism metabolism, and immune response. Interestingly, 7 families and 21 genera were significantly correlated with the level of inflammatory expression in rat lungs (*p* < 0.05). Moreover, there was a significantly negative correlation between the content of acetic acid and six varieties of bacteria, *Sporobacter*, *Marvinbryantia*, *Eubacterium*, *Prevotella*, *Romboutsia*, and *Blautia* (*p* < 0.05). In addition, a significantly positive correlation between the concentration of acetic acid and *Collinsella* was observed (*p* < 0.05).

### 3.4. Changes in SCFAs production

**Figure 5:**
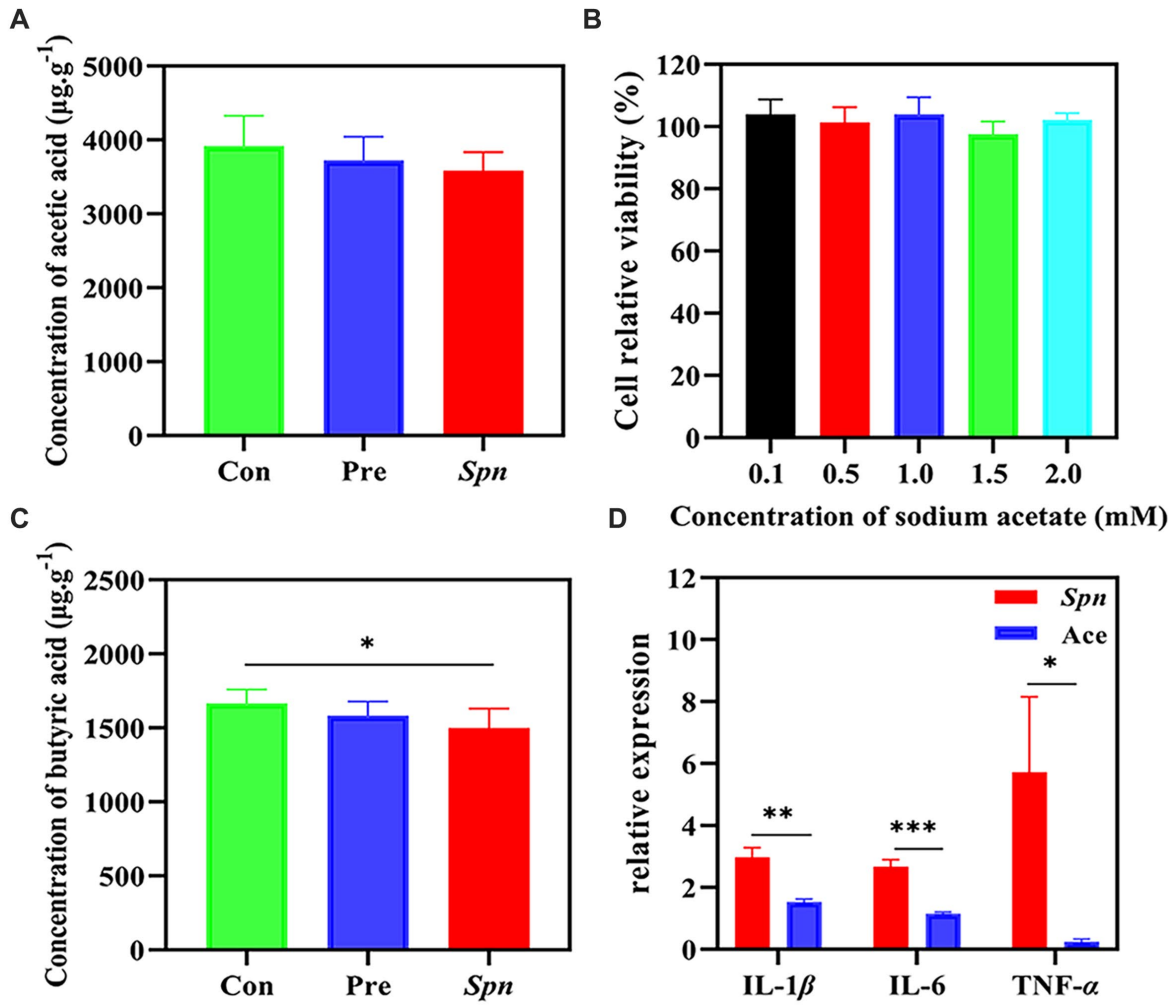
The concentration of acetic acid **(A)** and butyric acid **(B)** in feces. The cellular activity of BEAS-2B cells infected by *Spn* **(C)** and the protective effect of acetic acid on *Spn* infection **(D)**. The *p*-values * < 0.05, ** < 0.01, and *** < 0.001 indicate significant differences between groups.

In our experiments, *Spn* infection led to a decreasing trend in the concentrations of acetic acid (3585.3 ± 250.6 μg × g^−1^) in the intestine compared to healthy controls (3920.2 ± 407.9 μg × g^−1^), while GB24^T^ ameliorated the decreasing trend to some extent (3722.3 ± 322.6 μg × g^−1^; Figure 5A), although this change was not statistically significant. Meantime, the concentration of butyric acid in the *Spn* group (1499.6 ± 129.7 μg × g^−1^) was significantly lower than in the Con group (1734.2 ± 186.0 μg × g^−1^; *p* < 0.05). Furthermore, GB24^T^ supplementation resulted in a resurgence in the absolute concentration of acetic acid in the feces (1582.8 ± 95.5 μg × g^−1^) without statistically significant differences compared to the other two groups (Figure 5B). In addition, the concentration of acetic acid was 2065.0 ± 378.1 μg × mL^−1^ in the fermentation broth of GB24^T^ with OD600 = 2.3.

### 3.5. Acetic acid ameliorates *Spn*-induced pulmonary inflammation in BEAS-2B cells

Given the changes in acetic acid content and its close relationship with the gut microbial presentation, we further tested its protective effect against *Spn* infection causing inflammation by *in vitro* assays. As shown in Figures 5C,D, sodium acetate (0.1–2.0 mM) did not significantly affect cell viability (97.6% ± 4.1–104.0% ± 4.8%). *Spn* infection raised the expression of IL-1β, IL-6, and TNF-α by 2.978 ± 0.307, 2.679 ± 0.211, and 5.720 ± 2.434 folds in the Con group, respectively. GB24^T^ strain nasal supplementation significantly reversed the elevation of IL-1β (1.536 ± 0.085), IL-6 (1.158 ± 0.050), and TNF-α (0.247 ± 0.087) caused by *Spn* infection in BEAS-2B cells (*p* < 0.05).

## 4. Discussion

Bacteria rarely exist alone under natural conditions. Selective colonization of certain typical microbial communities is the mode of survival of most bacteria, and the swarming mode of survival determines the inevitable emergence of inter-bacterial communication and complex interactions. The mutual inhibition and even killing of bacteria have been of interest to the scientific community since the first isolation of antibiotics from *Streptomyces* (Peterson et al., 2020). This biological competitive stress, which occurs among bacteria and exists under almost all major phylum, is of great significance and has not only been shown to contribute to the stability of microbial communities but also to provide new ideas for treating and preventing some bacterial infectious diseases. The antagonistic ability of GB24^T^ against *S. pneumoniae* was close to cefditoren (minimal inhibitory concentration ≥ 1.0 μg × mL^−1^ had zone diameters of 16–24 mm) (Kelly et al., 1999), suggesting that GB24^T^ has the potential to be used in the development of novel prevention measures for *Spn* infections. A study in 2020 revealed that the mouse respiratory symbiont *L. murinus* could not only inhibit *Spn* growth *in vitro* by lactic acid fermentation but also prevent *Spn* colonization during respiratory dysregulation (Yildiz et al., 2020). In general, antagonism between bacteria often occurs due to competition for nutritional resources and production of released antimicrobial peptides and proteins (Garcia-García-Bayona and Comstock, 2018). In *in vitro* experiments, GB24^T^ exhibited some ability to produce acetic acid, which has been reported to antagonize the growth of *Streptococcus* (Mohammed et al., 2019).

*Spn* infection of the body is capable of releasing various inflammatory components, including peptidoglycan, pneumolysin, hydrogen peroxide, and many secreted proteins, some of which are not only already pro-inflammatory in their own right but also synergize with each other and finally induce inflammation through various inflammatory cascades, including the TLR pathway, chemokine/cytokine cascade, complement cascade, and coagulation cascade (Loughran et al., 2019). IL-1, IL-6, and TNF-α are important pro-inflammatory cytokines released by macrophages upon stimulation by bacteria or bacterial products and play an important role in the adaptive immune response of the body (Tang et al., 2021). The presence of these cytokines facilitates the host’s defense against microbial infections (Dinarello, 2000). On the other hand, however, an excessive pro-inflammatory response has been shown to trigger diseases such as gastrointestinal disorders (Mendes et al., 2019) and Parkinson’s disease (Lin et al., 2019). In this study, *Spn* infection caused a significant increase in IL-1β, IL-6, and TNF-α, suggesting that the *Spn* infection model was successfully established. Furthermore, GB24^T^ significantly reduced the gene expression levels of IL-1β, IL-6, and TNF-α, alleviating the structural disorder and congestion of the alveolar cavity caused by Spn infection. Combined with the good inhibitory performance of GB24^T^ organisms against *Spn* in the inhibition assay, it was assumed that the above-mentioned results were directly caused by the direct inhibitory effect of GB24^T^ on the colonization and growth of *Spn* in rat lungs, which can ultimately ameliorate the inflammatory damage by reducing *Spn* colonization. To confirm this conjecture, *Spn* colonization in rat lung tissues was further examined by qPCR, and the results showed that a higher density of *Spn* colonized the lung tissues in the *Spn* group, indicating that the exposure method of nasal inhalation could allow *Spn* to enter the deep airways and colonize the lung tissues smoothly. In addition, the density of *Spn* colonization in the lung tissues of the Pre group was lower and almost equal to that of the Con group, suggesting that GB24^T^ airway supplementation could efficiently reduce *Spn* burden in the lung tissues of rats. Normally, *Spn* colonization is usually manifested as adhesion to respiratory epithelial cells, which is mainly dependent on the binding of *Spn* to N-acetylglucosamine on the surface of epithelial cells and mediated by a series of cell wall surface proteins, including *Spn* surface adhesin A (PsaA). When local inflammation occurs in the organism or when inflammatory factors such as IL-1 and TNF accumulate, *Spn* can be internalized into cells to achieve viable bacterial invasion (Bogaert et al., 2004). The exact rationale for the reduction of *Spn* colonization capacity by GB24^T^ in this study was not clear, and follow-up experiments need to be designed to focus on the specific effects of GB24^T^ on *Spn* adhesion to cells.

Study addressing the gut–lung axis have shown that dysbiosis of the gut microbiota is one of the risk factors for numerous respiratory diseases (Zhang et al., 2020). In the present study, results of high-throughput sequencing of rat feces illustrated that GB24^T^ nasal intake increased the abundance of *Lactobacillus* in the intestine while reducing *Lachnospiraceae*, an opportunistic pathogen associated with impaired lung function (Bowerman et al., 2020). As a classical probiotic, *Lactobacillus* has been associated with respiratory infections, able to enhance the body’s resistance to *Spn* infections and avoid excessive inflammatory damage by promoting an increase in IgA cells in the intestine and airways and regulating TNF-α and IL-10 balance (Villena et al., 2011). Its increased numbers seen after GB24^T^ inhalation provide some explanation for the improvement of pulmonary inflammatory damage by GB24^T^ explanation. Furthermore, GB24^T^ intake in this study caused a significant decrease in *α* diversity of the rat intestinal microbiota compared to the *Spn* group, which could be attributed to the reduced abundance of some potentially pathogenic bacteria, such as *Ruminococcus* (De Filippis et al., 2021) that can participate in the pro-inflammatory response, *Clostridium* that trigger diarrhea (Krishna and Chopra, 2021), and *Helicobacter* that are associated with *H. pylori* infection (De Brito et al., 2019). At the phylum level, *Spn* infection resulted in a decreasing trend in the ratio of Bacillota to Bacteroidota, and the trend has also been observed in the gut of a rat model of acute lung injury induced by tracheal injection of lipopolysaccharide (LPS) by Tang et al. (2022). Currently, studies on intestinal microorganisms have revealed different roles played by Bacillota and Bacteroidota in inflammatory diseases, with an increased abundance of Bacillota promoting the production of the anti-inflammatory factor IL-10, whereas the role of Bacteroidota is more reflected in promoting the expression of pro-inflammatory cytokines (Wang et al., 2022). This finding explains the diminished inflammatory damage in rat lungs that occurs after GB24^T^ reverses the trend of decreasing the ratio of Bacillota to Bacteroidota. At the family level, *Spn* infection caused a significant decrease in *Lactobacillaceae* compared to the Con group, followed by enrichment after GB24^T^ intake, again indicating that *Lactobacillus* might be an important functional microorganism for the protective effect of GB24^T^ against *Spn* infection.

As one of the important metabolites of the intestine (Miller and Wolin, 1996), SCFAs are significant modulators of immune and inflammatory responses in infection (Ranjbar et al., 2021). Some studies conducted on SCFAs in recent years have revealed their beneficial applications in various respiratory diseases. Among them, butyric acid can ameliorate airway allergic inflammation (Theiler et al., 2019), while acetic acid and propionic acid can enhance the killing of *Spn* by alveolar epithelial cells through the NLRP3 inflammasome combined with glycolysis-HIF-1α axis and PI3K/Akt/mTOR signaling pathway, respectively, and ameliorate LPS-induced alveolar epithelial-mesenchymal transition (EMT) (Chen et al., 2020; Machado et al., 2022). Acetic acid is the most abundant organic acid in SCFAs, accounting for approximately 60–75% of the total SCFAs (Parada Venegas et al., 2019). Previous studies have demonstrated that the secretion of SCFAs can activate free fatty acid receptors (FFARs) to play an inflammatory regulatory role, and the sensitivity of FFARs to SCFAs determines the efficiency of the involvement of SCFAs in the inflammatory response. It has been suggested that the sensitivity of FFARs to SCFAs is not high, therefore limiting the regulation of inflammatory responses by SCFAs. Among the three FFARs, acetic acid and propionic acid preferentially bind to FFAR2, while butyric acid binds to FFAR3 to a greater extent. Outside the intestine, the high predominance of acetic acid allows it to successfully activate FFAR2 and, thus, participate in the regulation of inflammation (Bolognini et al., 2016; Milligan et al., 2017; Hoving et al., 2018).

SCFAs have been shown to migrate from the intestine to the lung along the gut–lung axis to form an anti-inflammatory environment (Dang and Marsland, 2019), although there are limited reports of SCFAs being detected in lung tissue. Furthermore, SCFAs have shown benign effects in a range of respiratory infectious diseases, such as promoting phagocytosis and killing *Klebsiella pneumoniae* by alveolar macrophages and enhancing lung resistance to viruses following respiratory syncytial virus (RSV) infection (Galvão et al., 2018; Antunes et al., 2019). *Spn* infection in this study caused a downward trend in the concentration of both acetic and butyric acids in the feces of rats, and the trend was restored after GB24^T^ intervention, although this resurgence was not statistically significant. Moreover, *in vitro* experiments confirmed that acetic acid was effective in alleviating inflammatory damage caused by *Spn* infection. The above-presented results suggest that acetic acid is an important metabolite in the defense of GB24^T^ against *Spn* infection.

## 5. Conclusion

We showed that GB24^T^ organisms not only inhibited the growth of *Spn* but also alleviated inflammatory damage caused by *Spn* infection by adjusting the composition of rat intestinal microbiota and increasing the secretion of SCFAs. Therefore, GB24^T^ is capable of alleviating *Spn* infection. Further study should be performed to investigate whether GB24^T^ is safe for the host and the ideal way to utilize it.

## Data availability statement

The datasets presented in this study can be found in online repositories. The names of the repository/repositories and accession number(s) can be found below: https://www.ncbi.nlm.nih.gov/, PRJNA908648.

## Ethics statement

The animal study was reviewed and approved by Southern Medical University Experimental Animal Ethics Committee (SMUL 2021099).

## Author contributions

TQ: methodology, investigation, data curation, writing-original draft, and writing-review and editing. TY and YL: investigation. JW and YJ: supervision. GZ: supervision, writing-original draft, and writing-review and editing.

## Funding

This work was supported by the National Science Foundation of China (NSFC 82173476).

## Conflict of interest

The authors declare that the research was conducted in the absence of any commercial or financial relationships that could be construed as a potential conflict of interest.

## Publisher’s note

All claims expressed in this article are solely those of the authors and do not necessarily represent those of their affiliated organizations, or those of the publisher, the editors and the reviewers. Any product that may be evaluated in this article, or claim that may be made by its manufacturer, is not guaranteed or endorsed by the publisher.

## Footnotes

^1^ https://www.bioincloud.tech/
