# Supplementary material for: Roseicella aerolata GB24T from bioaerosol attenuates Streptococcus pneumoniae-introduced inflammation through regulation of gut microbiota and acetic acid
Source: Front Microbiol. 2023 Jul 20;14:1225548. doi: 10.3389/fmicb.2023.1225548 (PMC10397393; doi:10.3389/fmicb.2023.1225548)
Supplement: Supplementary file 1 [file Data_Sheet_1.docx]

Supplementary Materials and Methods

*Roseicella aerolata* GB24^T^ from bioaerosol attenuates *Streptococcus pneumoniae*-introduced inflammation through regulation of gut microbiota and acetic acid

Tian Qin*, Ting Yu, Yuqi Liu, Jiguo Wu, Yunxia Jiang, Guoxia Zhang

*** Correspondence:**

Prof. Guoxia Zhang, E-mail: guoxiazhang@smu.edu.cn

Dr. Yunxia Jiang, E-mail: jiangyxia@126.com

# Supplementary Figures and Tables

## Supplementary Figures


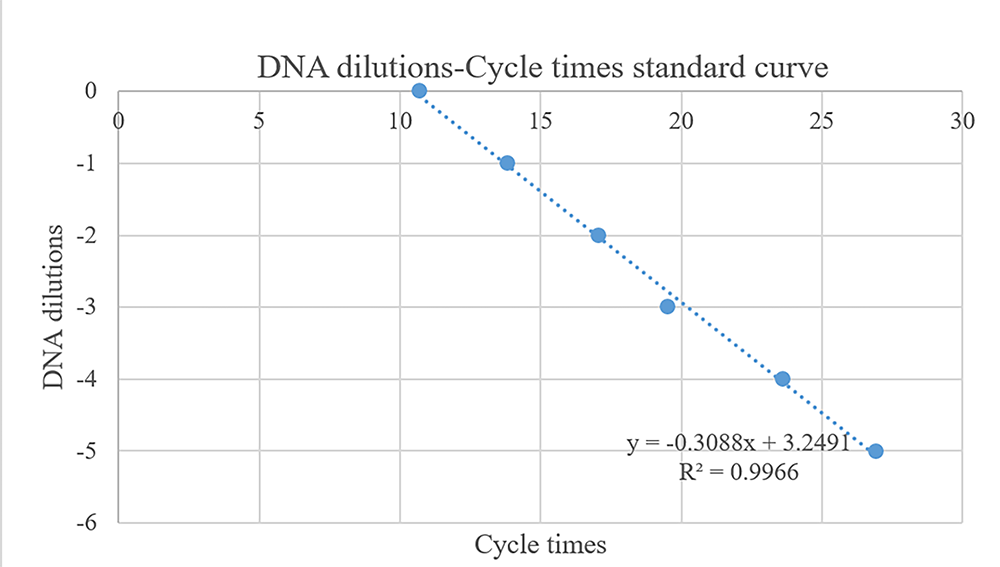


**Supplementary Figure 1.** Th**e** scatter plot between the concentration of DNA (C_DNA_) and qPCR cycle times (Ct). The *P*-values **<0.01 and ***<0.001 indicate significant differences between groups.

**
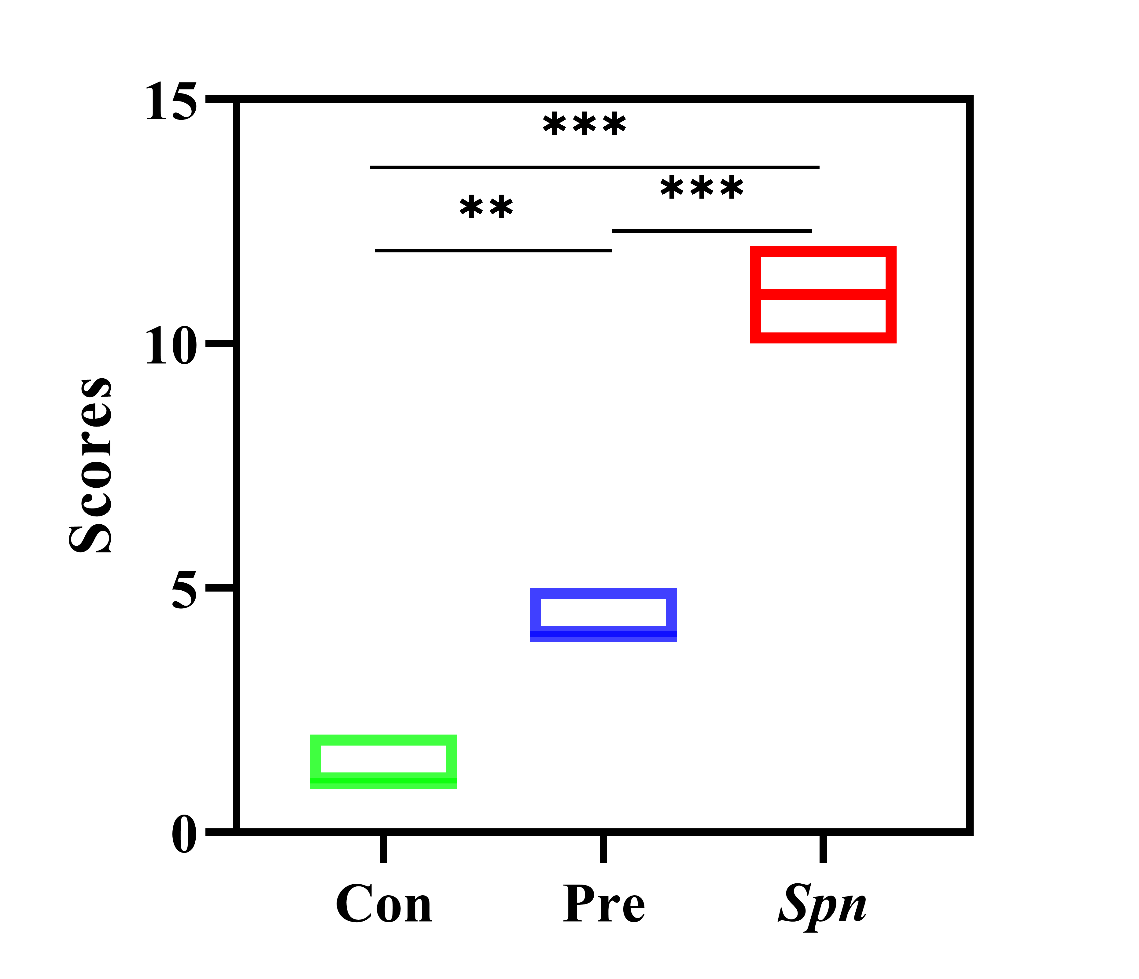
**

**Supplementary Figure 2.** Histopathological scores of lungs.


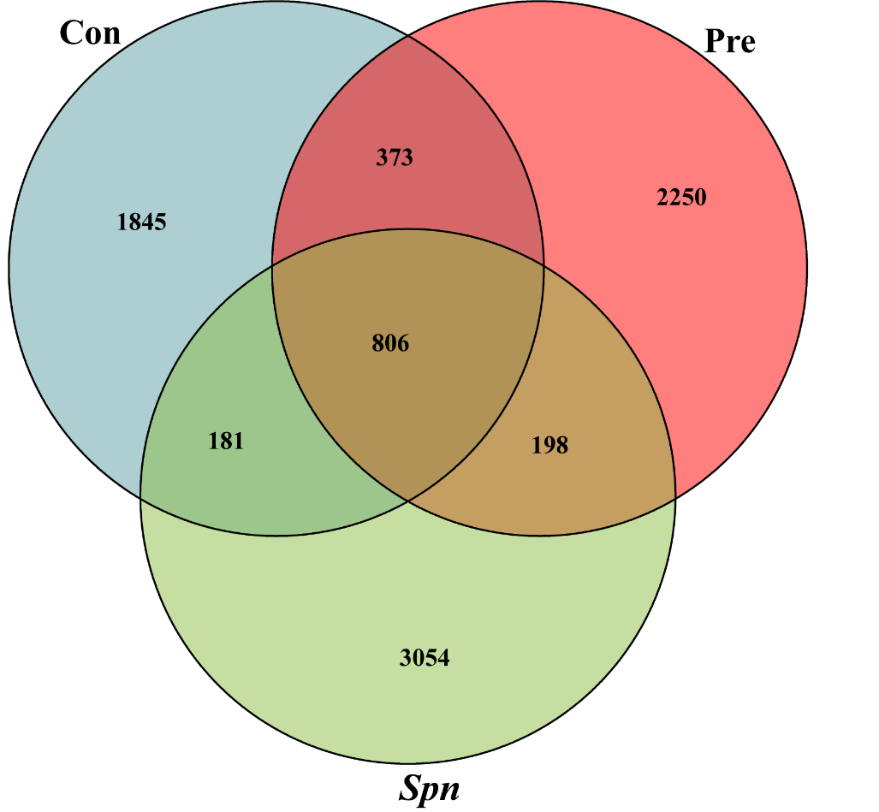


**Supplementary Figure 3.** Venn diagram based on OTUs. 3 circles represent different groups and the overlap represents common taxa. **
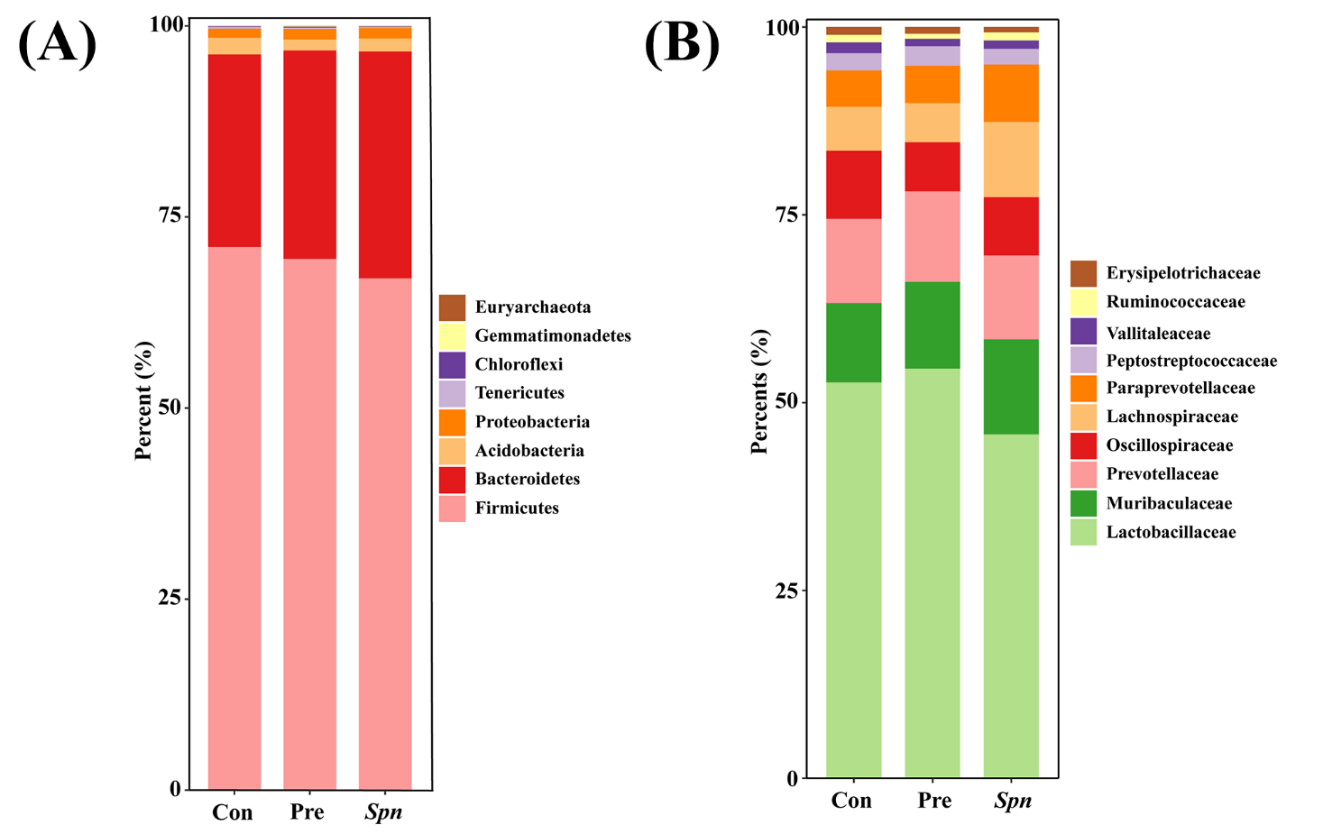
**

**Supplementary Figure 4.** Relative abundance at the phylum (A) and family (B) levels.

## Supplementary Tables

Supplementary Table 1. Cell grouping and treatments

| Group | Treatment 1 (1.5 h) | Treatment 2 (2.0 h) |
| --- | --- | --- |
| Control (Con) | DMEM medium | 2 mL DMEM medium |
| *Spn* | DMEM medium | *Spn* (MOI=20) |
| GB24^T^: *Spn* = 1:1 (1:1) | GB24^T^ (MOI=20) | *Spn* (MOI=20) |
| GB24^T^: *Spn* = 2:1 (2:1) | GB24^T^ (MOI=40) | *Spn* (MOI=20) |

Supplementary Table 2. Histopathological scoring criteria

| Histopathological changes | The severity and corresponding score |
| --- | --- |
| Alveolar cavity hemorrhage | No obvious lesions, 0; Mild, 1; Moderate, 2; Severe, 3; Extremely severe, 4 |
| Bleeding of lung tissue |  |
| Inflammatory cell infiltration |  |
| Alveolar wall deformation |  |

Supplementary Table 3. The primers for real-time qPCR

| Genes |  | Suquence (5’-3’) |
| --- | --- | --- |
| GAPDH (human) | Forward | GCACCGTCAAGGCTGAGAAC |
|  | Reverse | TGGTGAAGACGCCAGTGGA |
| IL-1*β* (human) | Forward | ATGATGGCTTATTACAGTGGCAA |
|  | Reverse | GTCGGAGATTCGTAGCTGGA |
| IL-6 (human) | Forward | ACTCACCTCTTCAGAACGAATTG |
|  | Reverse | CCATCTTTGGAAGGTTCAGGTTG |
| TNF-*α* (human) | Forward | CCTCTCTCTAATCAGCCCTCTG |
|  | Reverse | GAGGACCTGGGAGTAGATGAG |
| GAPDH (rat) | Forward | CTGAACGGGAAGCTCACTGG |
|  | Reverse | TCCGATGCCTGCTTCACTAC |
| IL-1*β* (rat) | Forward | GGCTTCCTTGTGCAAGTGTC |
|  | Reverse | AGTCAAGGGCTTGGAAGCAA |
| IL-6 (rat) | Forward | TCACTGTGCGTTGCAAACAGTGTC |
|  | Reverse | ATACCACAAGGTTGGCAGGTGGAT |
| TNF-*α* (rat) | Forward | ATGGGCTCCCTCTCATCAGT |
|  | Reverse | GCTTGGTGGTTTGCTACGAC |
| *lytA* | Forward | TCTTACGCAATCTAGCAGATGAAGC |
|  | Reverse | GTTGTTTGGTTGGTTATTCGTGC |

# Supplementary methods

According to the manufacturer’s protocols, microbial DNA was extracted from fecal samples using the E.Z.N.A.® soil DNA kit (Omega Bio-Tek, Norcross, GA, USA). The V3–V4 hypervariable regions of the bacterial 16S rRNA gene were amplified with primers 338F (5’-ACTCCTACGGGAGGCAGCAG-3’) and 806R (5’-GGACTACHVGGGTWTCTAAT-3’) by thermocycler PCR system (GeneAmp 9700, ABI, USA). The resulting PCR products were extracted from a 2% agarose gel, purified using the AxyPrep DNA gel extraction kit (Axygen Biosciences, Union City, CA, USA), and quantified using QuantiFluor™-ST (Promega, USA) according to the manufacturer’s protocol. Purified amplicons were pooled in equimolar and paired-end sequenced (2 × 300) on an Illumina MiSeq platform (Illumina, San Diego, USA). QIIME2 was used for bioinformatics analysis.
